# Short-Term Effect of Pitavastatin Treatment on Glucose and Lipid Metabolism and Oxidative Stress in Fasting and Postprandial State Using a Test Meal in Japanese Men

**DOI:** 10.1155/2013/314170

**Published:** 2013-12-10

**Authors:** Hirokazu Kakuda, Junji Kobayashi, Mio Nakato, Noboru Takekoshi

**Affiliations:** ^1^Kakuda Clinic, Takamatsu Na15-1, Kahoku, Ishikawa 929-1215, Japan; ^2^Department of General Medicine, Kanazawa Medical University, 1-1 Daigaku Uchinada, Kahoku, Ishikawa Prefecture 920-0293, Japan; ^3^Kanazawa Medical University, 1-1 Daigaku Uchinada, Kahoku, Ishikawa Prefecture 920-0293, Japan

## Abstract

*Introduction.* The objective of this study was to clarify how pitavastatin affects glucose and lipid metabolism, renal function, and oxidative stress. *Methods.* Ten Japanese men (average age of 33.9 years) were orally administered 2 mg of pitavastatin for 4 weeks. Postprandial glucose, lipoprotein metabolism, and oxidative stress markers were evaluated at 0 and 4 weeks of pitavastatin treatment (2 mg once daily) with a test meal consisting of total calories: 460 kcal, carbohydrates: 56.5 g (226 kcal), protein: 18 g (72 kcal), lipids: 18 g (162 kcal), and NaCl: 1.6 g. Metabolic parameters were measured at 0, 60, and 120 minutes after test meal ingestion. *Results.* After administration of pitavastatin, serum total cholesterol, low-density lipoprotein cholesterol, apolipoprotein B, arachidonic acid, insulin, and adjusted urinary excretion of uric acid decreased, whereas creatinine clearance (*C*_Cr_) and uric acid clearance (*C*_UA_) increased. And postprandial versus fasting urine 8-hydroxydeoxyguanosine remained unchanged, while postprandial versus fasting isoprostane decreased after pitavastatin treatment. Next, we compared postprandial glucose and lipid metabolism after test meal ingestion before and after pitavastatin administration. Incremental areas under the curve significantly decreased for triglycerides (*P* < 0.05) and remnant-like particle cholesterol (*P* < 0.01), while those for apolipoprotein E (apoE), glucose, insulin, and high-sensitivity C-reactive protein remained unchanged. *Conclusion.* Pitavastatin improves postprandial oxidative stress along with hyperlipidemia.

## 1. Introduction

It has been generally recognized that postprandial hyperglycemia and hyperlipidemia are highly related to the development of atherosclerosis [1–5]. Hyperglycemia is known to damage vascular endothelial cells, increase oxidative stress, promote the expression of adhesion molecules, and inhibit Nitric Oxide (NO) production [6]. Remnant lipoprotein, an important component of postprandial hyperlipidemia, promotes foam cell formation of macrophages and proliferation of smooth muscle cells [7]. A very recent study on a large number of subjects demonstrated that remnant cholesterol was a causal risk factor for ischemic heart disease [8].

Lipid-lowering drugs, such as statins, fibrates, and ezetimibe are considered to be useful for the treatment of postprandial hyperlipidemia [9–15]. Pitavastatin, a member of the medication class of statins, has been available in the market in Japan since 2003. It has been well recognized that this statin is markedly effective in reducing low-density lipoprotein cholesterol (LDL-C), triglycerides (TG), and increasing high-density lipoprotein cholesterol (HDL-C), while it is scarcely metabolized by hepatic drug-metabolizing enzymes cytochrome P450 (CYP) [16–21]. As a consequence, pitavastatin is most likely to be appropriate for patients with metabolic syndrome with high LDL, low HDL, and diabetes mellitus. To the best of our knowledge, however, there are few studies on the effect of pitavastatin on postprandial hyperlipidemia [22–24].

In addition, there is no previous study on the effect of pitavastatin on oxidative stress markers in postprandial states.8-Hydroxydeoxyguanosine (8-OHdG) and isoprostane are important markers for oxidative stress [25, 26]. Isoprostanes are chemically stable, free radical-catalyzed products of arachidonic acid (AA) that are structural isomers of conventional prostaglandins [27].

With this background, in this study we investigated the effect of pitavastatin treatment on glucose and lipoprotein metabolism and oxidized stress markers in the postprandial state using a mixed meal consisting of fat, glucose and proteins, and an established test meal [28] for the evaluation of both postprandial hyperglycemia and hyperlipidemia.

## 2. Material and Methods

### 2.1. Study Subjects

Japanese men, who agreed to undergo pitavastatin treatment and mixed meal test, were involved in this study (*n* = 10; age 33.9 ± 10.1 years; body height 172.0 ± 4.3 cm; body weight 80.2 ± 25.3 kg; body mass index (BMI) 27.0 ± 8.3 kg/m^2^; waist circumference 88.5 ± 18.9 cm). None of them had received medication.

### 2.2. Sample Collection

The subjects were not allowed to eat anything after 8 pm the day before blood sampling. Smoking was not allowed on the day of the examination. They were not allowed to intake alcohol two days before the examination. On the morning of the day of examination, the study subjects did a complete urination and took 100 mL of water at 8 am, and then did a second urination right before blood sampling (at 0 min), followed by the oral ingestion of a test meal (JANEF E460F18, Q.P. Co., Tokyo, Japan) consisting of total calories 460 kcal, carbohydrates 56.5 g (226 kcal, 49.1%), Protein 18 g (72 kcal, 15.7%), lipids 18 g (162 kcal, 35.2%), and NaCl 1.6 g. The subjects spent 10 min of taking E460F18 with 120 mL of water and underwent blood and urine sampling at 60 minutes and 120 minutes. Pitavastatin administration (2 mg once daily) started from the next day for 4 weeks. After 4 weeks of pitavastatin administration, a meal tolerance test using E460F18 was again conducted exactly the same way it was done before starting pitavastatin.

### 2.3. Measurement of Metabolic Parameters

Cholesterol and triglycerides were measured by the enzyme method. HDL-C and LDL-C were measured by direct homogeneous assay methods using detergents. Quantification of remnant-like particles cholesterol (RLP-C) was conducted by a method using an immune-separation technique (Otsuka Pharmaceutical Co., Ltd.).

A total of 5 *μ*L of plasma was mixed with 300 *μ*L of lipoprotein separation medium consisting of a Sepharose gel suspension to which monoclonal antibodies directed against apoB-100 (JIH) and apoA-1 had been attached. The separation medium was gently mixed for 120 min, and thereafter allowed to settle for another 15 min. The cholesterol content (RLP-C) was assayed enzymatically in the supernatant with an autoanalyzer. Serum Insulin levels were measured using enzyme-linked immunosorbent assay kits (Cosmo Bio Co., Ltd., Tokyo, Japan). Urinary 8-Hydroxydeoxyguanosine (8-OHdG) and levels were measured using enzyme-linked immunosorbent assay kits (Cosmo Bio Co., Ltd., Tokyo, Japan). Urinary isoprostane levels were measured using enzyme-linked immunosorbent assay kits (Funakoshi Co.,Ltd., Tokyo, Japan).

High-sensitivity C-reactive protein (hsCRP) levels were determined using an enzyme-linked immunosorbent assay kit (Dade Behring Marburg GmbH, Tokyo, Japan).

### 2.4. Analysis of Postprandial Lipid Metabolism

Postprandial metabolism was quantified by calculating the incremental area under the curve (iAUC) using each value at 0 minutes, 60 minutes, and 120 minutes after E460F18 loading. Results are presented as mean ± sd. Differences between parameters obtained before and after pitavastatin therapy were evaluated by paired Student's *t*-test analysis.

Parameters that did not distribute normally (BMI, triglycerides, insulin, and HOMA-R) were analyzed by The Wilcoxon signed-rank test. Dunnett's test was used for the multiple comparisons.

### 2.5. Ethics and Consent

This work was carried out in accordance with The Code of Ethics of the World Medical Association (Declaration of Helsinki) for experiments involving humans.

Informed consent was obtained from all of the participants. The institutional review board in Kanazawa Medical University Hospital approved the experimental protocol, and all of the subjects provided informed consent to participate in the study.

## 3. Results

### 3.1. Changes in Metabolic Parameters in Fasting States before and after Pitavastatin Treatment

Table 1 shows changes in each indicator in fasting states before (at 0 weeks) and at 4 weeks after pitavastatin administration (2 mg/day). Four-week-pitavastatin treatment was not associated with changes in body mass index at all. There were reductions in total cholesterol (TC) LDL-C (direct method) and apolipoprotein B (apo B). There were no significant changes in fasting TG and RLP-C. Among 4 types of fatty acid, there was a significant reduction in arachidonic acid (AA). There was no significant change in plasma glucose but there was a reduction in fasting insulin.

No significant changes were observed in hsCRP and urine 8-OHdG. There were considerable increases in *C*_cr_, *C*_UA_, and R (*C*_UA_/*C*_cr_ × 100%) and a decrease in adjusted urinary excretion of uric acid.

### 3.2. Changes in Time-Course of Lipid Parameters after Oral Loading of a Test Meal E460F18 before and after Pitavastatin Treatment

**Figure 1:**
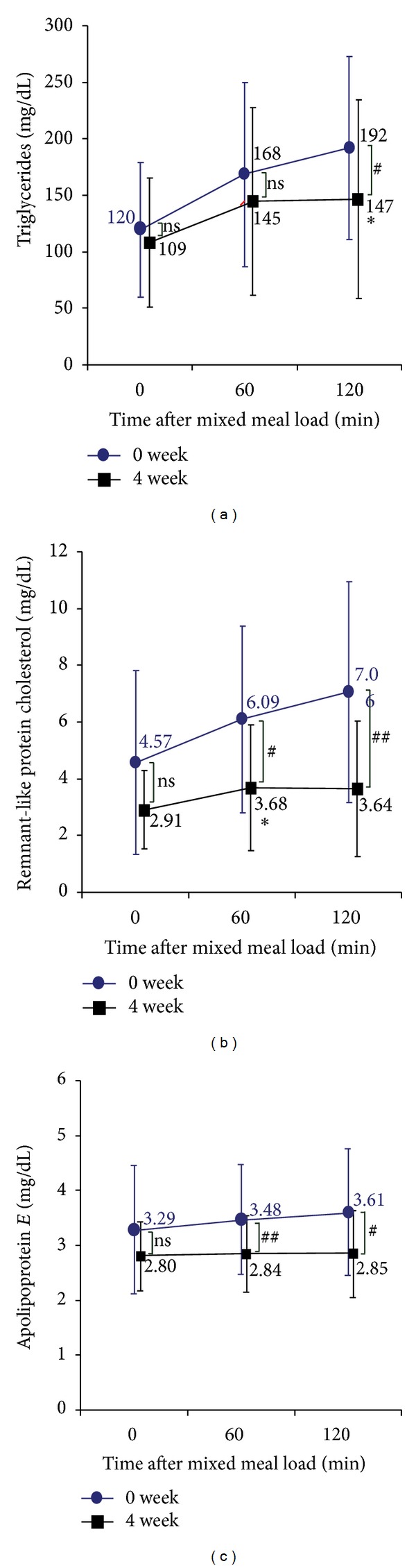
Changes in each parameters related to lipids after oral loading of E460F18 (a),triglycerides; (b),remnant-like protein cholesterol; (c), apolipoprotein E; Data are presented as mean ± standard deviation. ns, not significant.

**Figure 2:**
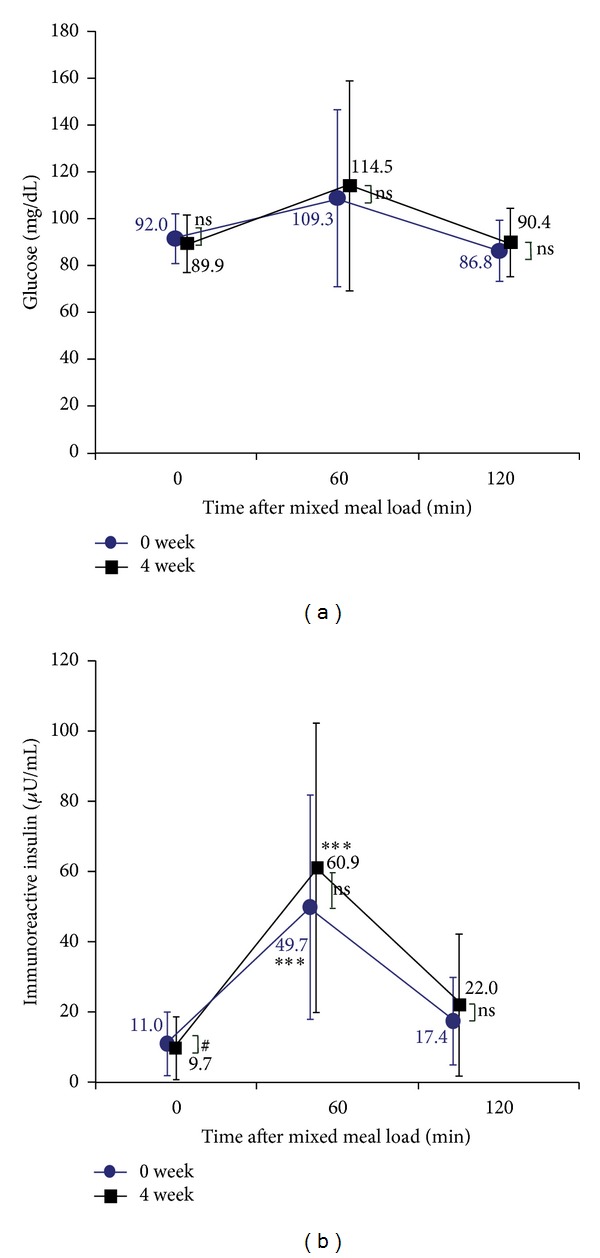
Changes in each parameters related to glucose after oral loading of E460F18 (a), Glucose; (b), immunoreactive insulin; Data are presented as mean ± standard deviation. ns, not significant.

**Figure 3:**
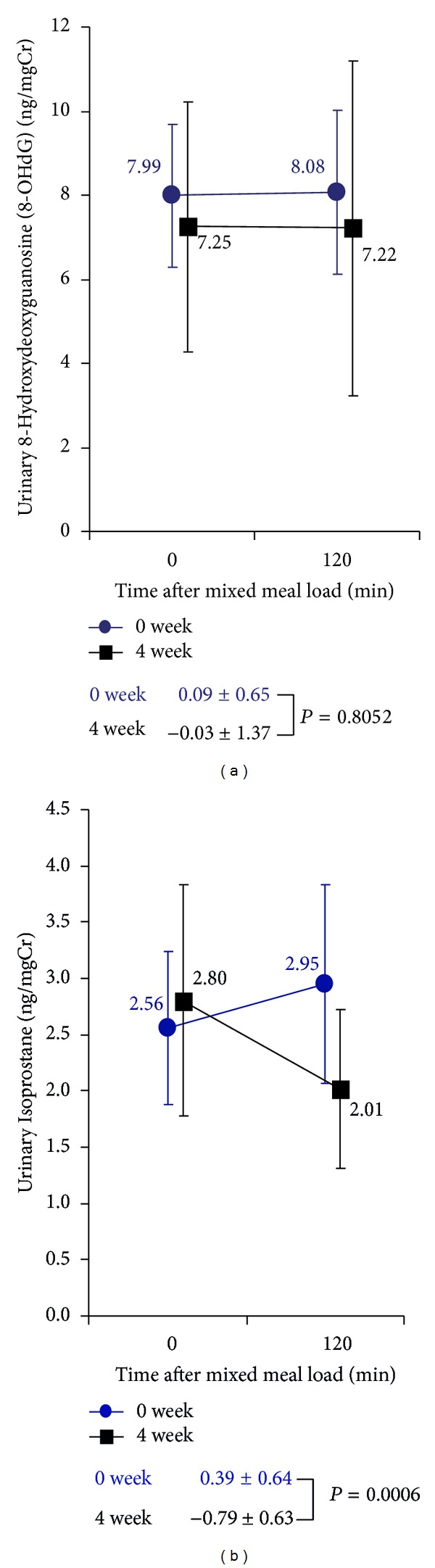
Changes in oxidative stress markers after oral loading of E460F18 (a), urinary 8-Hydroxydeoxyguanosine (8-OHdG); (b), urinary Isoprostane; Data are presented as mean ± standard deviation. ns, not significant.

Figure 1 through Figure 3 show differences in the time course of metabolic parameters after E460F18 loading between before (at 0 weeks) and after (at 4 weeks) pitavastatin treatment.  Table 2 shows changes in iAUC between 0 weeks and 4 weeks. TG at 120 minutes after the test meal loading significantly decreased at 4 weeks versus 0 weeks. Related to this, pitavastatin treatment was associated with significant reductions in iAUC for TG (Table 2). Similarly, and more importantly, RLP-C at 60 min and 120 min and iAUC for RLP-C after E460F18 loading were significantly decreased at 4 weeks versus 0 weeks (Figure 1 and Table 2). Further, apo E at 60 min and 120 min after the load was significantly reduced, but the reduction of its iAUC was not significant (Figure 1 and Table 2). With respect to postprandial glucose metabolism, immunoreactive insulin, and glucose at 60 min and 120 min after E460F18 loading did not significantly change at 4 W versus 0 W (Figure 2).

### 3.3. Changes in Time-Course of Parameters for Oxidative Stress Index after Oral Loading of a Test Meal E460F18 before and after Pitavastatin Treatment

We also analyzed changes in oxidative stress index after pitavastatin treatment. There was no difference in postprandial changes in urine 8-OHdG between before and after pitavastatin treatment, whereas the postprandial increase in urine isoprostanes observed before pitavastatin treatment tended to decrease after pitavastatin treatment (Figure 3).

## 4. Discussion

In this study we clarified that pitavastatin treatment produced considerable improvement in markers related to oxidative stress as well as those related to lipid metabolism including RLP-C in postprandial states in Japanese men with abdominal obesity. This is the first study investigating pitavastatin effects on oxidative stress markers in postprandial states after test meal loading as well as fasting states.

For fasting states, pitavastatin treatment was associated with significant decreases in fasting insulin levels as well as decreases in fasting TC, LDL-C, and apoB.

For postprandial states, pitavastatin treatment was associated with marked reductions in TG-iAUC and more so in RLP-iAUC. Several studies show that plasma RLP-C, not TG, is an independent risk for the development of atherosclerotic disease [29, 30].

There are several studies on the effect of atorvastatin, another strong statin, on postprandial hyperlipidemia and the mechanisms of its improvement have been considered to be an inhibition of the production of very low-density lipoprotein (VLDL) caused by sustained inhibition of cholesterol synthesis in the liver [31] and increased lipoprotein lipase (LPL) activity leading to hydrolysis of TG-rich lipoproteins [11]. Parhofer et al. have suggested that atorvastatin promoted the uptake of chylomicron remnants by the liver through LDL receptors, leading to the improvement of postprandial lipid metabolism in normolipidemic subjects [13] and hypertriglyceridemic subjects [14]. It is also shown that atorvastatin inhibits the production of apolipoprotein CIII, leading to increases in LPL activity [12]. Compared with atorvastatin, there appears to be much less information available so far on the effect of pitavastatin on postprandial hyperlipidemia and the mechanism by which it exerts its effectiveness. Saiki et al. [32] have reported using preadipocyte 3T3L1 that among pravastatin, simvastatin, atorvastatin, and pitavastatin, pitavastatin was the strongest to increase the activity of LPL. Morikawa et al. have found that when using Hep G2 cells the effect of pitavastatin on the induction of LDL receptor was stronger compared with other statins [33]. Based on these findings, we suggest that in the current study pitavastatin increased the number of LDL receptors in the liver along with an increase in LPL activity, which in turn could cause the improvement of postprandial lipid metabolism. Moreover, other mechanisms could contribute to the improvement of postprandial lipid metabolism by pitavastatin. It has been suggested that pitavastatin decreases the expression of mRNA of microsomal triglyceride transfer protein (MTTP) from the liver in an animal model of postprandial hyperlipidemia [24, 34].

A postprandial increase in oxidative stress is considered to be an important contributor to the impairment of endothelial function, leading to the development of atherosclerosis and there is a study showing the close link between increases in both TG and that in oxidative stress [35]. Indeed in the current study, we found that increases in urine isoprostanes, an oxidative stress marker, in the postprandial states observed before pitavastatin treatment decreased after pitavastatin treatment. To our knowledge, this is the first study suggesting that pitavastatin treatment inhibits the production of isoprostanes during postprandial states.

The mechanism by which this occurred has not been clarified yet, but there is a study suggesting the contribution of adiponectin to the reduction of isoprostanes during atorvastatin treatment [36]. Also, pitavastatin treatment is reported to be associated with increased adiponectin in hyperlipidemic patients with type II diabetes [37].

We also analyzed the effect of pitavastatin treatment on changes in renal function. Among them the present findings that *C*_cr_ increased after the treatment is compatible with our previous report [38]. We presume that this effect of pitavastatin could be related to the induction of endothelial NO synthase (eNOS) by this drug [39], since NO synthesis inhibition has been associated with decreases in renal plasma flow (RPF) and glomerular filtration rate (GFR) [40]. These observations suggest that pitavastatin may be a suitable drug for treatment of hyperlipidemia with chronic kidney disease (CKD).

The imitations of this study are that the sample size is small and study was done in a single arm. However, we conducted measurements of a wide range of metabolic markers at several time points in postprandial states as well as fasting states before and after pitavastatin treatment. Also it should be noted that to the best of our knowledge there have been only 2 clinical studies on the effect of pitavastatin on postprandial metabolism [22, 23], both of which mainly focused on the prevention of postprandial endothelial dysfunction after pitavastatin treatment.

In conclusion, the present findings suggest that in men with abdominal obesity, pitavastatin improves oxidative stress and renal function along with hyperlipidemia, especially in regards to remnant metabolism, in the postprandial state, contributing to the prevention of the development of atherosclerosis. It is also suggested that pitavastatin improves renal function.

## Figures and Tables

**Table 1:** Fasting metabolic parameters at 0 and 4 weeks after pitavastatin.

	0 Weeks	4 Weeks	*P *
Body mass index (kg/m^2^)	23.4 (21.3–29.2)	23.7 (21.7–29.4)	0.557
Total cholesterol (mg/dL)	197.9 ± 23.6	156.5 ± 16.2	0.0001
Low-density lipoprotein cholesterol (mg/dL)	121.5 ± 22.4	81.9 ± 15.1	<0.0001
High-density lipoprotein cholesterol (mg/dL)	56.6 ± 10.1	56.8 ± 9.4	0.8321
Triglycerides (mg/dL)	98.5 (78.0–138.8)	89.5 (63.0–132.8)	0.287
Remnant-like particle cholesterol (mg/dL)	4.57 ± 3.25	2.91 ± 1.38	0.1239
FFA (mEq/L)	0.52 ± 0.19	0.51 ± 0.23	0.8926
Apolipoprotein AI (mg/dL)	138.9 ± 13.7	141.6 ± 12.6	0.3207
Apolipoprotein B (mg/dL)	89.6 ± 23.0	66.6 ± 13.3	0.0004
Apolipoprotein E (mg/dL)	3.29 ± 1.17	2.80 ± 0.63	0.0735
Arachidonic acid (*μ*g/mL)	184.9 ± 32.2	166.1 ± 31.5	0.0485
Eicosapentaenoic acid (*μ*g/mL)	60.2 ± 39.2	76.8 ± 55.5	0.3319
Docosahexaenoic acid (*μ*g/mL)	121.0 ± 55.7	113.9 ± 53.8	0.6055
Glucose (mg/dL)	92.0 ± 10.6	89.9 ± 12.2	0.4502
Insulin (*μ*U/mL)	5.9 (4.9–16.3)	5.9 (2.8–15.7)	0.028
HOMA-R	1.28 (1.12–3.80)	1.26 (0.56–3.47)	0.322
High sensitivity CRP (mg/L)	0683 ± 0791	0436 ± 0566	0.187
Urinary 8OHdG (ng/mgCr)	7.99 ± 1.70	7.25 ± 2.97	0.1882
Urinary isoprostane (ng/mgCr)	2.56 ± 0.68	2.80 ± 1.03	0.4423
Urinary isoprostane production rate (ng/kg/hour)	2.32 ± 1.24	2.65 ± 1.19	0.4894
Serum creatinine (mg/dL)	0.87 ± 0.09	0.86 ± 0.12	0.8143
eGFR (mL/min/1.73 m^2^)	84.6 ± 12.1	85.9 ± 14.8	0.5168
Albumin/creatinine ratio (mg/gCr)	6.82 ± 7.49	5.39 ± 5.79	0.4383
*C*_Cr_ (mL/minute)	98.1 ± 34.4	134.2 ± 31.0	0.0431
Serum uric acid (mg/dL)	5.97 ± 1.17	5.82 ± 0.76	0.4786
*C*_UA_ (mL/minute)	5.1 ± 2.4	7.8 ± 1.9	0.0072
*C*_UA_/*C*_Cr_ (%)	5.1 ± 1.7	6.0 ± 1.7	0.0409
Uric acid excretion (mg/gCr)	353.4 ± 157.8	405.3 ± 129.9	0.2385
Adjusted urinary excretion of uric acid (mg/kg/hour)	1.75 ± 0.73	0.98 ± 0.76	0.0378

Values are shown as mean ± standard deviation. BMI, triglycerides, insulin, and HOMA-R are shown as median and interquartile range. HOMA-R: homeostasis model assessment ratio; 8OHdG: 8-Hydroxydeoxyguanosine; eGFR: estimated glomerular filtration rate; *C*_Cr_: creatinine clearance; *C*_UA_: uric acid clearance.

**Table 2:** Changes in incremental area under the curve (iAUC) for each metabolic parameter after E460F18 loading at 0 and 4 weeks of pitavastatin treatment.

	0 week	4 week	*P *
Triglycerides (mg/dL)	3885 (2565–6480)	2745 (1883–4508)	0.007
Remnant-like particle cholesterol (mg/dL)	166 ± 125	68 ± 85	0.0012
Apolipoprotein E (mg/dL)	21 ± 29	4 ± 13	0.1333
Glucose (mg/dL)	882 ± 2175	1491 ± 2216	0.2289
Insulin	2342 (1202–3083)	2664 (1441–5439)	0.093
*C*_Cr_ (mL/minute)	5048 ± 7975	6058 ± 4660	0.7752
Albumin creatinine ratio	−68 ± 194	−194 ± 312	0.2424
*C*_UA_ (mL/minute)	536 ± 317	534 ± 292	0.9920
Adjusted urinary excretion of uric acid (mg/kg/hour)	−88 ± 55	−37 ± 53	0.0442

Values are shown as mean ± standard deviation. *C*_Cr_: creatinine clearance; *C*_UA_: uric acid clearance.

iAUC for triglycerides and insulin are shown as median and interquartile range.
